# Frenectomía lingual con láser ND:YAG. reporte de caso

**DOI:** 10.21142/2523-2754-1102-2023-158

**Published:** 2023-06-29

**Authors:** Adriana Pares Perfetti, Natacha Valentina Guada Melet, José Alberto Castillo Páez

**Affiliations:** 1 Facultad de Odontologia, Departamento de Prostodoncia y Oclusion de la Universidad de Carabobo. Valencia, Venezuela. adrianaparesp@gmail.com, natachaguada@hotmail.com Universidad de Carabobo Facultad de Odontologia Departamento de Prostodoncia y Oclusion Universidad de Carabobo Valencia Venezuela adrianaparesp@gmail.com natachaguada@hotmail.com; 2 Facultad de Odontologia, Departamento de Estomatoquirurgica de la Universidad de Carabobo. Valencia, Venezuela. josecastillo031285@gmail.com Universidad de Carabobo Facultad de Odontologia Departamento de Estomatoquirurgica Universidad de Carabobo Valencia Venezuela josecastillo031285@gmail.com

**Keywords:** frenectomía lingual, anquiloglosia, láser Nd:YAG, lingual frenectomy, ankyloglossia, Nd:YAG laser

## Abstract

La anquiloglosia es considerada una anomalía congénita cuyo tratamiento indicado es la frenectomía, técnica quirúrgica que consiste en la remoción del tejido que une la lengua con el piso de la boca. Esta técnica permite al paciente una mejoría considerable de las limitaciones causadas por dicha anomalía, como las dificultades en la succión, pronunciación, masticación, y la dificultad para tocar el labio inferior con la punta de la lengua. En la actualidad, el uso de la tecnología láser y sus beneficios en la odontología moderna permite la realización de procedimientos quirúrgicos libres de sangrado, con muy poco dolor e inflamación de los tejidos, y con un tiempo de recuperación mucho menor a los necesarios con técnicas convencionales. En el presente caso clínico, se presenta una paciente de 12 años de edad con una anquilosis severa, por lo que se le indicó frenectomía lingual. Para ello, se utilizó un láser Nd:YAG de contacto, con lo que se logró una intervención quirúrgica con muy poco dolor, libre de sangrado y sin necesidad de sutura.

## Abstract (en)

Ankyloglossia is considered a congenital patology, whose treatment indicated is a frenectomy, this surgical technique consists in remove the tissue that joins the tongue with the floor mouth. This technique allows the patient a considerable improvement to limitations caused by this anomaly such as difficulties in suction, pronunciation, chewing and difficulty in touching the lower lip with the tip of the tongue. Currently the use of laser technology and its benefits in modern dentistry, allows the realization of surgical procedures free of bleeding, with very little pain and inflammation of the tissues and with a recovery time much lower than necessary with conventional techniques. The present clinical case, show a 12-year-old patient with severe ankyloglossia, lingual frenectomy was indicated. A contact Nd: YAG laser was used, achieving a surgical intervention with little pain, free bleeding and without suture.

## INTRODUCCIÓN

El frenillo lingual es una membrana mucosa situada en la parte ventral de la lengua y que la une con el piso de la boca ^1^^,^^2^. Se caracteriza por ser un sólido cordón, que se inicia en la cara inferior de la lengua. El extremo anterior del frenillo lingual se asienta en la cara lingual de la mandíbula y en el borde de la arcada dentaria, es decir, entre los incisivos centrales inferiores ^2^. Histológicamente, el frenillo lingual está compuesto por un tejido conjuntivo rico en fibras colágenas y elásticas, con algunas fibras musculares, vasos sanguíneos y células engrosadas, y recubierto por un epitelio pavimentoso estratificado ^3^ (Tabla 1).

**Tabla 1:** Clasificación clínica del frenillo corto ^8^

Leve	Moderado	Severo
Frenillo corto que no interfiere con las funciones de la lengua: succión, deglución, masticación, fonación.	Frenillo corto que dificulta la alimentación; posteriormente, causa alteraciones mínimas en el lenguaje, el periodonto y la posición de los dientes.	Lengua fusionada con el piso de la boca; hay dificultad para la deglución y la succión. Se pueden observar problemas periodontales. Marcada dificultad en el lenguaje.

Algunas veces, su inserción puede ser anormalmente corta y dificultar los movimientos de la lengua, la fonación y la deglución, entre otros. Esta anomalía es conocida como anquiloglosia ^4^ (Tabla 2). Los síntomas más comunes son imprecisión de la articulación del habla, cambio de fonema por otro o con distorsión, pequeña apertura de la boca durante el habla, imprecisión o ineficacia de los movimientos de la lengua en movimientos aislados, forma de corazón en el ápice de la lengua cuando es protraída, lengua con poca protrusión o aún con protrusión que lleva su ápice hacia abajo, lengua con postura en el piso de la boca, dificultades para mover la punta de la lengua, dificultad de succión durante el amamantamiento, masticación ineficiente y deglución con alteración por dificultad de acoplamiento de la lengua en el paladar duro, entre otras ^5^. Además, ella interfiere también en el proceso de cepillado y, por consiguiente, favorece el riesgo de acumulación de placa, instalación de inflamación tisular y retracción de encías ^6^^,^^7^.

**Tabla 2:** Clasificación de la anquiloglosia según el rango de lengua libre ^9^

Clase I	Leve	12 a 16 mm de lengua libre
Clase II	Moderada	8 a 11 mm de lengua libre
Clase III	Severa	3 a 7 mm de lengua libre
Clase IV	Completa	< 3 mm de lengua libre

A pesar de ser una entidad clínica bastante reconocida, la anquiloglosia en niños menores de 1 año representa un desafío en cuanto a su diagnóstico para los odontólogos. La anquiloglosia parcial es la más común. Esa anormalidad dificulta los movimientos de la lengua, principalmente en la pronunciación de ciertas consonantes y diptongos labiodentales ^7^^,^^8^.

La IATP (International Affiliation of Tongue-Tie Professionals) lo define como un tejido embrionario residual situado en la línea media de la lengua, entre su cara inferior y el suelo de la boca que restringe el movimiento normal de la lengua ^1^. Es importante destacar que la anquiloglosia presenta una incidencia variable del 0,02% al 11% de la población de recién nacidos y una relación hombre mujer de 3:1 en países de América Latina ^2^^,^^10^. En el caso específico de Venezuela, no se consiguieron estadísticas soportadas por fuentes fidedignas sobre la prevalencia de esta anomalía.

La anquiloglosia es considerada una anomalía congénita cuyo tratamiento indicado es la frenectomía. Esta técnica quirúrgica consiste en la remoción del tejido que une la lengua con el piso de la boca ^11^. Este procedimiento era convencionalmente realizado con escalpelo, hasta que fueron introducidos el electrocauterizador y, más tarde, el láser como instrumentos para su realización, la cual tiene el fin de devolver la funcionalidad del área afectada mediante la extirpación de dicho repliegue anatómico, a partir del empleo de instrumentación manual o láser ^12^^,^^13^.

Debido al reciente avance en la aplicación del láser y su uso de manera eficaz para el diagnóstico, la prevención y el tratamiento de la caries dental, así como para procedimientos mínimamente invasivos, la Academia Estadounidense de Odontología Pediátrica también recomienda el uso racional del láser de tejidos duros y blandos para diferentes procedimientos orales en bebés, niños y adolescentes ^14^^-^^16^.

Los procedimientos quirúrgicos convencionales pueden generar rechazo por parte del paciente, debido al tiempo de intervención prolongado y el curso del posoperatorio; sin embargo, en el campo odontológico actual, con el advenimiento del láser de alta intensidad y su aplicación en procedimientos quirúrgicos, se logra controlar estos factores con una técnica poco invasiva, menos dolorosa, efectiva, rápida, precisa y segura desde el punto de vista biológico, lo que genera mayor interés y expectativas en los pacientes ^15^^,^^16^.

La tendencia de la odontología es la incorporación de métodos menos invasivos para minimizar el dolor y las molestias durante y después del tratamiento. Por lo tanto, se cree que la terapia láser es una excelente opción, ya que tiene efectos beneficiosos para los tejidos irradiados, tales como la activación de la microcirculación, la producción de nuevos capilares, acción antiinflamatoria y efectos analgésicos, así como el crecimiento estimulante y regeneración celular ^15^^,^^16^.

El tratamiento de un paciente pediátrico con láser para procedimientos orales y dentales es beneficioso, ya que genera menos miedo para el niño y es mejor aceptado por los padres. Cuando el médico utiliza el láser para un procedimiento quirúrgico o pulpar, los niños cooperan más y también mejora el resultado del tratamiento. Se utiliza para la prevención de la caries, el diagnóstico temprano, la restauración de cavidades, el manejo de dientes traumatizados y procedimientos quirúrgicos orales menores en pacientes infantiles, y parece convertirse pronto en el estándar de oro en la práctica dental pediátrica ^16^.

El uso de láser de diodo en procedimientos de cirugía oral es beneficioso tanto para el paciente como para el odontólogo. La frenectomía lingual asistida por láser es fácil de realizar, con excelente precisión, menos incomodidad, menos tiempo de recuperación, mejor percepción del dolor posoperatorio y la posibilidad de no utilizar sutura debido a su excelente hemostasia ^7^^,^^17^^,^^18^.

En caso de realizar una frenectomía con láser de alta intensidad, se busca que sea un procedimiento resectivo y de colocación de la inserción del frenillo lingual utilizando las ventajas que ofrece la tecnología láser, a diferencia de la cirugía convencional con bisturí, mediante un corte preciso en poco tiempo, con reducción de la hemorragia e inflamación en los tejidos, asepsia y analgesia, lo cual se debe a que los vasos sanguíneos cuyos diámetros son menores al del haz del rayo láser se vaporizan ^14^^,^^18^.

El objetivo del presente reporte de caso es presentar el manejo quirúrgico del frenillo lingual utilizando láser Nd:YAG (láser de alta intensidad) en la reubicación de la inserción del frenillo con la finalidad de mejorar la función de la lengua, para reestablecer la adecuada movilidad, fonación y deglución.

## REPORTE DEL CASO CLÍNICO

El paciente y su representante consintieron la publicación del presente reporte de caso. Sus aspectos bioéticos fueron revisados y aprobados por la Comisión de Bioética de la Facultad de Odontología de la Universidad de Carabobo, Valencia, Venezuela.

**Figura 1:**
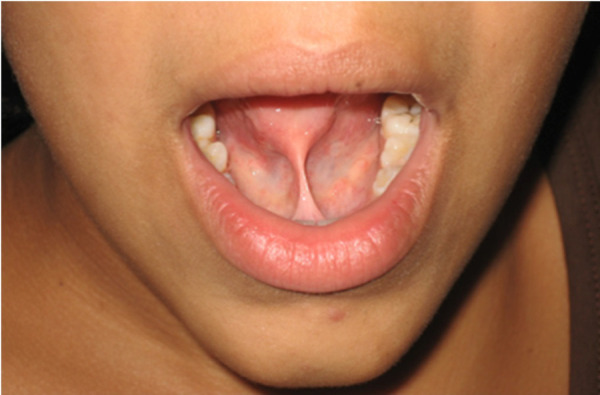
Examen clínico preoperatorio. Se observa el grado de anquilosis del frenillo lingual.

En el presente caso clínico, se describe la condición oral de un paciente femenino de 12 años, que acude a la consulta por presentar dificultad en la succión, pronunciación, masticación, y para tocar el labio inferior con la punta de la lengua, así como elevarla en dirección al paladar, debido a la gran anquilosis lingual clase III, según Kotlow. Por ello se recomienda la frenectomía lingual con láser de alta intensidad como tratamiento de esta anomalía (Fig. 1).

**Figura 2:**
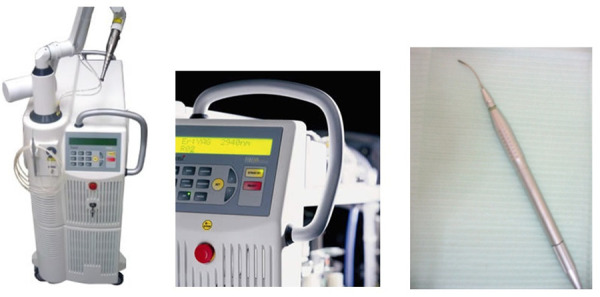
Equipo Fidelis Plus (Fotona) utilizado para realizar el tratamiento

El equipo utilizado en este caso fue Fidelis Plus, un láser Nd:YAG, el cual cuenta con una longitud de onda de 1,064 µm, transmitido mediante una fibra óptica de contacto de 300 µm de diámetro, estableciendo como parámetros de funcionamiento 5 W de poder y una frecuencia de 15 Hz (Fig. 2).

**Figura 3:**
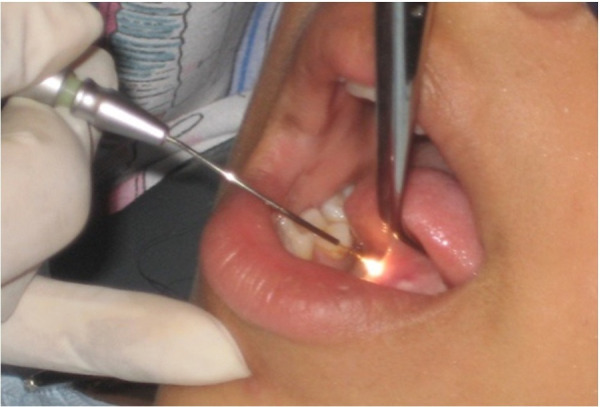
Acto quirúrgico de eliminación de frenillo utilizando láser Nd:YAG

Se colocó anestesia infiltrativa y se procedió a traccionar la lengua. Seguidamente, se realizó el corte desde el punto de inserción más cercano a la punta de la lengua; por el gran tamaño del frenillo, el corte se continuó paralelo a la cara ventral, realizando movimientos de contacto intermitente hasta eliminar por completo el tejido fibroso que ataba la lengua al piso de la boca, devolviéndole la movilidad fisiológica normal y respetando la desembocadura de la glándula sublingual. El tiempo de duración de la cirugía fue de 17 minutos (Fig. 3).

**Figura 4:**
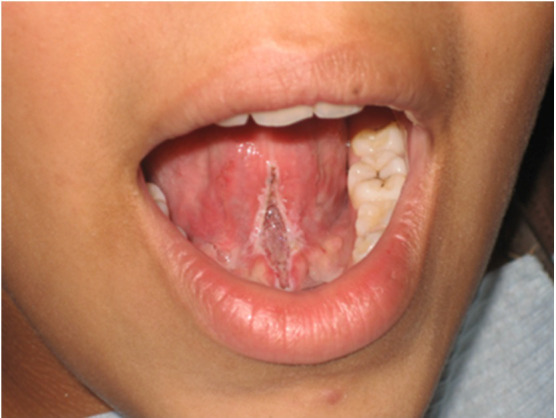
Posoperatorio inmediato donde se puede observar ausencia de inflamación y sangrado. No se requiere sutura y la cicatrización es más rápida.

Durante el procedimiento, se mantuvo un campo operatorio libre de sangrado (Figs. 4 y 5). Cabe destacar la reacción positiva del paciente debido a la ausencia de dolor intraoperatorio. Se indicó la realización de ejercicios fonoaudiológicos luego de 24 horas de realizada la cirugía para evitar una posible recidiva, así como tratamiento profiláctico y analgésico en caso de dolor o molestia.

**Figura 5:**
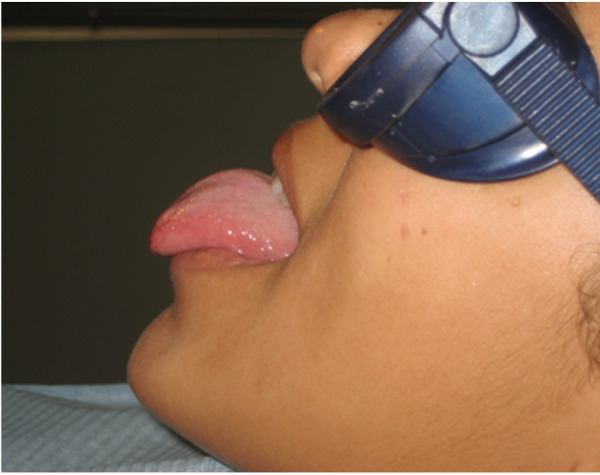
Finalizada la cirugía, se demuestra la movilidad de la lengua.

Se controló a la paciente a las 72 horas, y se observó la ausencia de dolor e inflamación en la zona tratada, así como se evidenció una disminución en la disfonía y una mayor posibilidad de movimientos linguales. Nuevamente, se controló a los siete días, se observó una cicatrización casi total y una gran mejora en la movilidad gracias a los ejercicios fonoaudiológicos.

## DISCUSIÓN

En el presente caso clínico, se utilizó el láser Nd:YAG por su capacidad para penetrar profundamente en el tejido, según lo afirman Yadav *et al*. ^19^, lo que lo hace ideal para procedimientos de tejidos blandos, como la frenectomía. Otros beneficios de usar este láser, según los autores mencionados, incluyen la reducción del dolor, la inflamación y la infección posoperatoria, para el procedimiento no se requiere sutura, la contracción resultante y la cicatrización son mínimas, y no hay necesidad de anestesia local ^16^^,^^17^^,^^19^.

La disminución en la percepción del dolor se puede atribuir a la coagulación de la proteína en la superficie de la herida que actúa como un vendaje biológico, sellando así los extremos de los nervios sensoriales ^16^^,^^19^. Además, el período posoperatorio fue cómodo, calificado así por la paciente, con ningún dolor indicado por el uso mínimo de analgésicos. Esto se debe a que el láser causa un daño colateral mínimo y también provoca el sellado de los linfáticos. Además, se forma un coágulo de fibrina sobre el sitio quirúrgico que lo protege de la irritación externa ^20^.

No se evidenció sangrado durante la cirugía. Así como lo describe el estudio de Patel *et al*., que registró significativamente menos sangrado intraoperatorio con frenectomía asistida por láser ^21^. Esto puede atribuirse a la coagulación de proteínas de tejidos blandos a alta temperatura de ablación tisular, que resulta en una reducción del sangrado en los márgenes del tejido ablacionado. Además, a alta temperatura, las paredes de los vasos sanguíneos se contraen y causan coagulación fototérmica ^15^^,^^16^^,^^22^.

Por lo descrito anteriormente, el estudio clínico indica que el láser de Nd:YAG puede considerarse una alternativa viable a las técnicas convencionales para el abordaje de la anquilosis mediante la frenectomía. Los láseres de alta intensidad tienen la ventaja de una mejor aceptación por parte del paciente, debido a la reducción de la percepción del dolor y el malestar posoperatorio. Además, se encuentra una reducción del sangrado intraoperatorio en comparación con el bisturí ^17^^,^^19^.

## CONCLUSIÓN

Como conclusión, se evidenció una incisión precisa, reducción del trauma quirúrgico intra y posoperatorio, y mejor cicatrización, por lo cual se observó clínicamente, en el control a las 72 horas, disminución del proceso inflamatorio y dolor, disminución del uso de anestésico local, así como campos operatorios limpios y sin sangrado.

Sin embargo, el alto costo y la necesidad de habilidad y entrenamiento del operador son limitaciones asociadas a esta técnica. De igual manera, se hace necesario realizar estudios a largo plazo con un tamaño de muestra mayor para establecer con mayor precisión la eficacia de la técnica láser en procedimientos quirúrgicos de tejidos blandos, específicamente en frenectomía.

## Fuente de financiamiento:

La presente investigación fue financiada por los autores
